# Exploring intrinsically disordered proteins in *Chlamydomonas reinhardtii*

**DOI:** 10.1038/s41598-018-24772-7

**Published:** 2018-05-01

**Authors:** Yizhi Zhang, Hélène Launay, Antoine Schramm, Régine Lebrun, Brigitte Gontero

**Affiliations:** 1 Aix Marseille Univ, CNRS, BIP, UMR 7281, IMM, 31 Chemin J. Aiguier, 13402 Marseille, Cedex 20 France; 2 Aix Marseille Univ, CNRS, AFMB, UMR 7257 Marseille, France; 3 Plate-forme Protéomique, Marseille Protéomique (MaP), IBiSA labeled, IMM, FR 3479, CNRS, B.P. 71, 13402 Marseille, Cedex 20 France

## Abstract

The content of intrinsically disordered protein (IDP) is related to organism complexity, evolution, and regulation. In the Plantae, despite their high complexity, experimental investigation of IDP content is lacking. We identified by mass spectrometry 682 heat-resistant proteins from the green alga, *Chlamydomonas reinhardtii*. Using a phosphoproteome database, we found that 331 of these proteins are targets of phosphorylation. We analyzed the flexibility propensity of the heat-resistant proteins and their specific features as well as those of predicted IDPs from the same organism. Their mean percentage of disorder was about 20%. Most of the IDPs (~70%) were addressed to other compartments than mitochondrion and chloroplast. Their amino acid composition was biased compared to other classic IDPs. Their molecular functions were diverse; the predominant ones were nucleic acid binding and unfolded protein binding and the less abundant one was catalytic activity. The most represented proteins were ribosomal proteins, proteins associated to flagella, chaperones and histones. We also found CP12, the only experimental IDP from *C*. *reinhardtii* that is referenced in disordered protein database. This is the first experimental investigation of IDPs in *C*. *reinhardtii* that also combines *in silico* analysis.

## Introduction

Some biologically active proteins have no well-defined tertiary structure in their native state and are known as intrinsically disordered proteins (IDPs) while other proteins possess structural elements with some disordered (flexible) regions (IDRs)^1–3^. It is well-known that the lack of protein structure is determined by the amino acid sequence^4^ and indeed, IDPs or IDRs have a biased amino acid composition. Compared to other proteins, they are enriched in charged and structure-breaking residues (Pro and Gly) and in Ala residues while they are depleted in hydrophobic and aromatic residues and have low content of Cys and Asn residues^5–10^.

Although proteins may have different conformations and be folded or unfolded depending on different conditions^11^, in IDPs, order-disorder transitions can be triggered by pH, temperature, redox potential, mechanical force, light exposure and various types of interactions. IDPs or IDRs are often the target of phosphorylation, ubiquitination, methylation, breakage of disulfide bridges and disorder-order transitions can result from these post- translational modifications (PTMs)^12,13^. Recently 4588 phosphoproteins and 115 protein kinases in *C*. *reinhardtii* were detected using phosphorylation and kinome enrichment strategies coupled to mass spectrometry but without considering intrinsic flexibility of these proteins^14^.

Because of their dynamic properties and flexibility allowing them to bind a wide range of partners, IDPs are often central hubs and play multiple roles in biological processes^2,13,15,16^. According to previous proteome-wide studies, intrinsic flexibility is widespread in all kingdoms of Life^17^, with eukaryotes having a significantly larger fraction of intrinsic disorder in their proteomes than prokaryotes^18^. The average content of flexible proteins is 3.8% in archaea, 5.7% in bacteria, and 18.9% in eukaryotes suggesting that increasing protein flexibility is related to the complexity of an organism^19^. Transcription factors containing IDRs are likely key factors contributing to the evolution of organismic complexity as they have important roles in the regulation of the cell cycle, division, differentiation and proliferation and in cell size^20,21^. IDRs in proteins, as well as the alternative splicing of their precursor mRNA and their phosphorylation, constitute a driving force in the evolution of complex multicellularity^22^. Flexibility or plasticity allows functional diversification and environmental responsiveness^23^ and since photosynthetic organisms are complex and require a high level of regulation to cope with their changeable environment, a large number of flexible proteins are expected within their proteome. However; only 51 IDPs from photosynthetic species are referenced in the database for disordered proteins^24,25^. This number is significantly lower than the 157 bacterial IDPs, the 62 IDPs from fungi and the 400 IDPs from vertebrates. This relatively low proportion of identified IDPs from photosynthetic organisms among the 804 IDPs of the DisProt database^25^ illustrates the lack of study of structural disorder on these organisms, and does not reflect the true proportion of IDPs within the different Life kingdoms.

In Plantae, two specific families of proteins relying on disorder for their functioning have been well described: the dehydrins including protein chaperones such as ERD10 and ERD14^26,27^ and the GRAS family^28,29^. Dehydrins play major roles under specific conditions including responses to abiotic stress including drought^28,30^ and GRAS proteins are involved in hormone responses. They are therefore critical for plant adaptation and survival^31^. Nevertheless, as mentioned above, only a few analyses of the global IDP content in photosynthetic organisms are available, and are based on bioinformatic search^32,33^. Experimental methods to identify the flexible proteins have been proposed and applied to other organisms^34^ including the bacterium, *Escherichia coli*, the yeast, *Saccharomyces cerevisiae*^35^ and the mouse^36^. In the higher plant, *Arabidopsis thaliana*, a systematic analysis of the seed phosphoproteome was performed using heat-treatment followed by phosphoaffinity chromatography to identify phosphorylated IDPs. This study showed that several late-embryogenesis-abundant (LEA) proteins and storage-like proteins were major components of the seed phosphoproteome^37^. While the characterization of the flexible proteins is the focus of numerous studies, experimental identifications of IDPs are still lacking and are thus needed to bring an added value to the set of bioinformatic data already available.

The eukaryotic green alga, *Chlamydomonas reinhardtii*, is a well-known biological model, and has been extensively studied and referred to as the photosynthetic yeast^38^. There are only a few IDPs reported from this green alga, such as the Chloroplast Protein (CP12), which forms a supramolecular complex with two key Calvin-Benson-Bassham (CBB) cycle enzymes, glyceraldehyde-3-phosphate dehydrogenase (GAPDH) and phosphoribulokinase (PRK)^39–42^. This protein regulates the association–dissociation of this complex, thereby allowing the CBB cycle to be inactive in the dark and active in the light, but has moonlighting activities^43^, for instance, chaperone function^44^ and metal ions binding^45^. Another IDP recently found in this alga is the Essential Pyrenoid Component 1 (EPYC1), a low complexity repeat protein that binds the ribulose-1, 5-bisphosphate carboxylase-oxygenase to form the pyrenoid matrix^46^.

While an entire proteome bioinformatic analysis has been performed for ten eukaryotes, including *C*. *reinhardtii*, providing a reliable collection of disorder annotations, statistics, and relevant disorder parameters from protein amino acid sequences^33^, these authors concluded that these results need to be confronted with experimental data.

To bring new information on amino acid compositions, cellular compartments and molecular functions of algal IDPs, we searched for flexible proteins from *C*. *reinhardtii* based on their heat-resistance property and we characterized them by mass spectrometry coupled to *in silico* approaches. We compared our experimental results to the whole proteome of *C*. *reinhardtii* using a bioinformatics analysis. This work will help to bring a conceptual breakthrough for an in-depth understanding of the molecular mechanisms of IDPs and their role in the cellular physiology of this alga.

## Results

### IDPs enrichment in *C*. *reinhardtii* extracts and their identification

**Figure 1:**
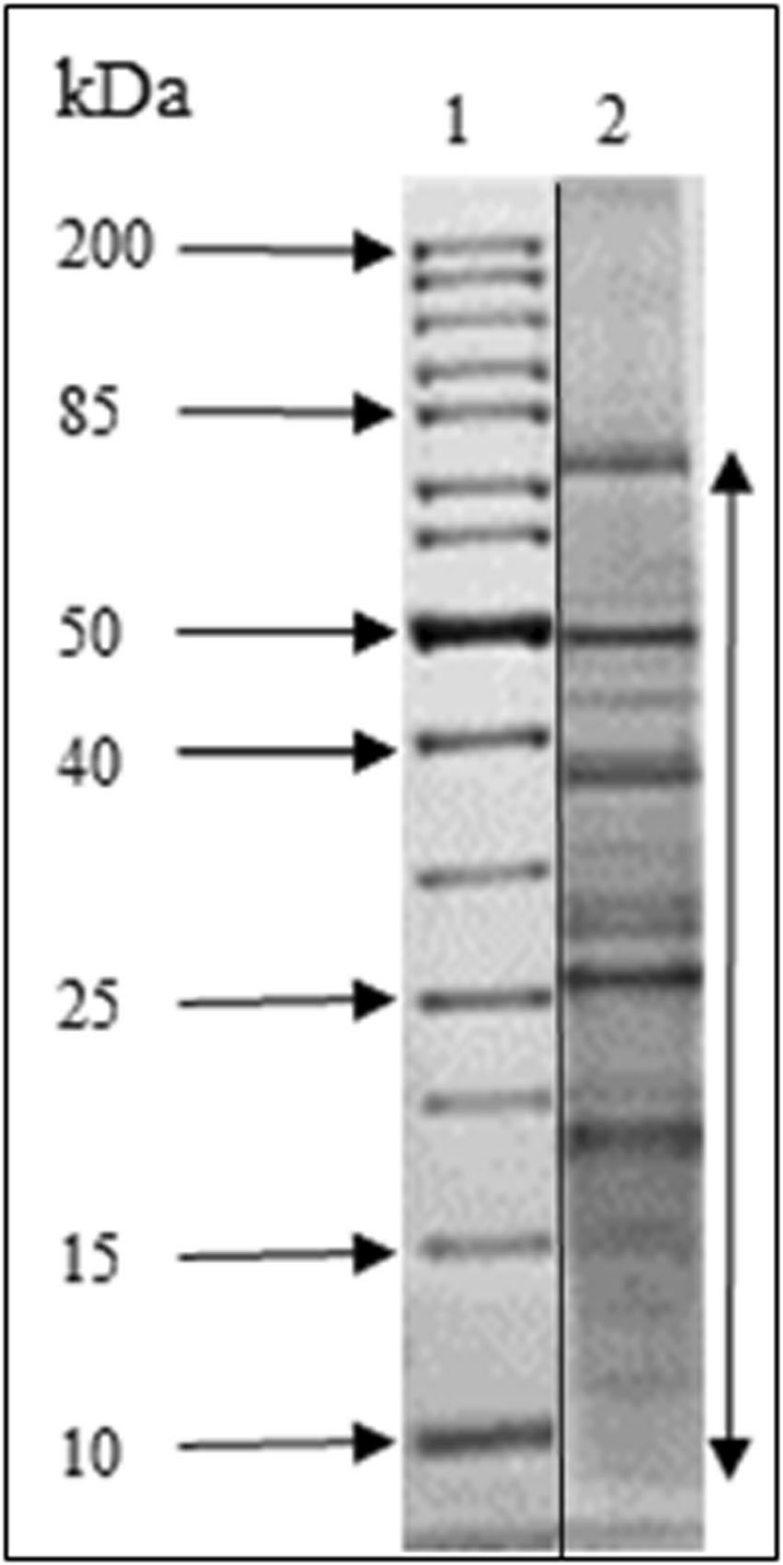
SDS-PAGE of heat-treated proteins from *C*. *reinhardtii*. 10 µg were loaded and separated on 12% polyacrylamide gels under denaturing conditions and stained with Coomassie Blue (Lane 2), molecular weight markers (Euromedex. unstained protein ladder, Lane 1). The two lanes came from different parts of the same gel (see Fig. S1). The most abundant bands in the range of molecular weight (M_w_) shown by the arrow were sliced and identified by mass spectrometry.

**Figure 2:**
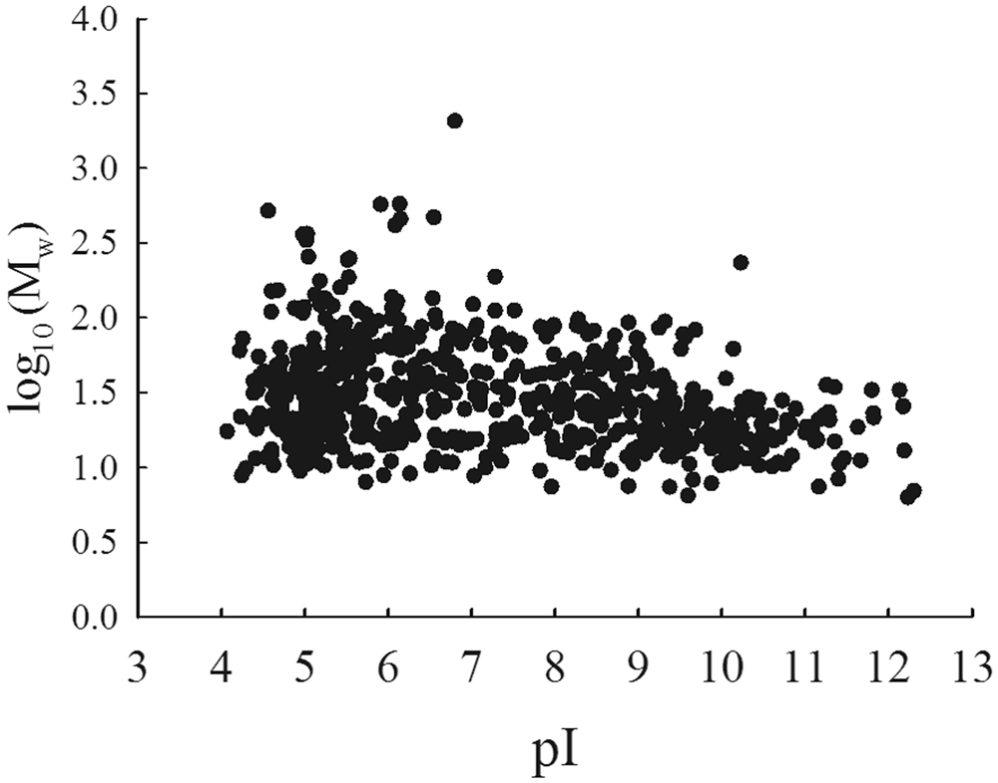
Distribution of isoelectric point (pI) value and molecular weight (M_w_) of 682 experimentally identified IDPs from *C*. *reinhardtii*. The pI and the M_w_ were theoretical and given by the mass spectrometry software.

IDPs or IDR-containing proteins are well-known to remain soluble under some critical conditions, such as extreme pH and temperature whereas globular proteins unfold, aggregate and precipitate. Therefore, to characterize proteins with flexibility in *C*. *reinhardtii*, proteins (about 2.4 mg) extracted from this alga were either acid- or heat-treated for at least 5 min. About 7% of the total proteins were heat-resistant while only 0.3 to 0.5% were acid-resistant. When heated for longer time, up to 1 h, the amount of proteins in the supernatant or soluble fraction did not change. To study as many experimental IDPs as possible, heat-treatment was chosen (Table 1). The heat-stable proteins (proteins remaining soluble after heat-treatment) expected to be IDPs or IDR-containing proteins were further analyzed by SDS-PAGE (Fig. 1 and Supplementary Fig. S1). The most intense bands were analyzed by liquid chromatography tandem mass spectrometry (LC-MS/MS) after trypsin digestion. We identified 791 heat-resistant proteins from NCBI database search (15313 proteins) and also searched against Phytozome v12.1 (19526 proteins). Among the 791 heat-resistant proteins, the sequence of 109 proteins was only partial and thus 682 proteins were analyzed further. These 682 proteins are listed in Supplementary Table S1. Their theoretical biophysical properties are: a broad range of isoelectric points (4 < pI* < *12) and of molecular masses with most proteins ranging within 10 to 200 kDa as expected from SDS-PAGE (Fig. 2). The most represented proteins were ribosomal proteins (57), proteins associated to flagella (33), chaperones (20) and histones (9). We also found the proteins that are biochemically well-characterized in details as being IDPs such as CP12 and EPYC1 or IDR-containing protein such as adenylate kinase 3.

**Table 1:** Heat and acid-treatments.

Treatment	Protein (mg)	% of putative IDPs
Control	2.38 ± 0.05	NA
5% TCA	0.007 ± 0.003	0.3
10% PCA	0.010 ± 0.005	0.5
98 °C, 1 h	0.164 ± 0.021	6.9
98 °C, 30 min	0.161 ± 0.023	6.8
98 °C, 5 min	0.159 ± 0.018	6.7

Content (mg) and percent of heat- or acid-resistant proteins in *C*. *reinhardtii*. Control corresponds to the total protein concentration in the samples before treatment. Values are means with standard error, n = 5. TCA and PCA stands for trichloroacetic acid and perchloric acid, respectively.

Even though proteases inhibitors were added, ten proteins (1.5% of the 682 heat-resistant proteins) were obviously degraded. These proteins were Type I polyketide synthase, Dicer-like protein, flagellar-associated protein, SNF2 superfamily protein, and hypothetical proteins (see Supplementary Table S1), and they have a high theoretical molecular mass, out of the range of the SDS-PAGE but their degraded fragments contain disordered residues. This is in agreement with the analysis by PONDR that showed that these large multi-domain proteins have a high proportion of disordered residues from 26 to 97%.

A side-observation concerns phosphorylation of 19 proteins. Indeed, since our approach was not aimed at enriching phosphorylated proteins, a few phosphorylated proteins were experimentally highlighted and are listed in Supplementary Table S2. We then searched how many proteins within the 682 proteins were phosphorylated using the phosphoproteome data from the literature^14^. We found 331 proteins corresponding to about 50% of the total proteins extracted (682) that were phosphorylated. The entire list of the 331 phosphorylated proteins can be found as Supplementary Table S3.

### *In silico* analysis of “experimentally found” IDPs

**Figure 3:**
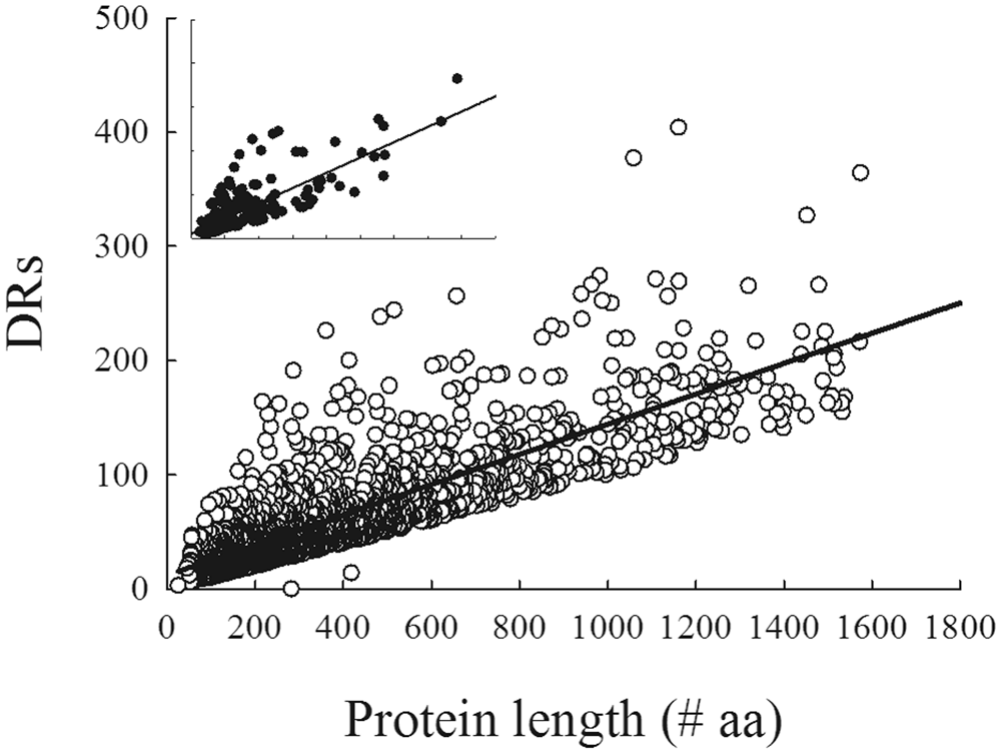
Relationship between disordered residues (DRs) and protein length. Open circles and full circles represent the 2152 IDPs analyzed from^33^ and the 299 experimental IDPs from this study (inset); in both case, only proteins with a percent disorder ≥10% were analyzed. A linear relationship between DR calculated from the disorder percent from Vincent and Schnell^33^ and protein length was found with a regression coefficient a = 0.17 and a correlation coefficient R^2^ = 0.6.

After analyzing the properties of the heat-resistant proteins, we specifically investigated their content of flexibility using bioinformatic analysis. Searching for flexible region higher than 30 consecutive amino acid residues as previously reported in the literature^10,47^, 506 proteins (74.1% of the 682 identified proteins, Table 2) were validated as flexible using PONDR while IUPred, DisEMBL and FoldIndex, validated 244 (35.7%), 101 (14.8%) and 260 (38.1%) proteins, respectively. Only 43 proteins were consistently selected by all the four predictors as each predictor relies on different philosophies: some are a priori algorithms and others are trained on existing datasets. Agreement among them therefore, should not be expected for all the proteins. We then modified our criteria and included proteins with the longest disorder length (LDR comprised between 10 and 30 residues) but with a percent of flexibility higher than 10. In that case, PONDR considered 98% (669/682) of the proteins to be IDPs confirming our experimental procedure. FoldIndex, DisEMBL and IUPred considered 498 (73%), 448 (66%), and 348 (51%) proteins to be IDPs, respectively. 299 proteins (44%) were selected as IDPs by all four software (Table 2). These results highlight the importance to combine many predictors and approaches to increase reliability of disorder analysis^47^. We also searched for the IDPs found in our study and for all predicted IDPs from a recent *in silico* approach that used other criteria for disorder^33^. After removing partial sequences, among the 9418 proteins left, we found 2152 IDPs that had a disorder percent higher than 10. Among these 2152 IDPs, 205 proteins from our experimental dataset with disorder percent higher than 10 were listed. The mean percentage of flexibility of this subset of proteins was 23% compared to 17.4% for the initial set of 2152 proteins. The relationship between the protein length and the disordered residues was analyzed for these two sets of IDPs. A linear relationship was observed, meaning that the longer the protein, the more the disordered residues (Fig. 3).

**Table 2:** Number of experimental proteins with the longest disordered region determined by different predictors.

Predictor	Length range				
	0~9	10~29	30~49	50~99	100<
PONDR	16 (2.3%)	160 (23.6%)	198 (29%)	181 (26.5%)	127 (18.6%)
IUPred	300 (44.1%)	138 (20.2%)	73 (10.7%)	95 (13.9%)	76 (11.1%)
DisEMBL	175 (25.8%)	406 (59.4%)	62 (9.0%)	33 (4.8%)	6 (0.9%)
FoldIndex	262 (38.3%)	160 (23.4%)	99 (14.6%)	111(16.3%)	50 (7.3%)

Percentage is given in parentheses taking into account a total of 682 proteins.

### Putative subcellular localization

**Figure 4:**
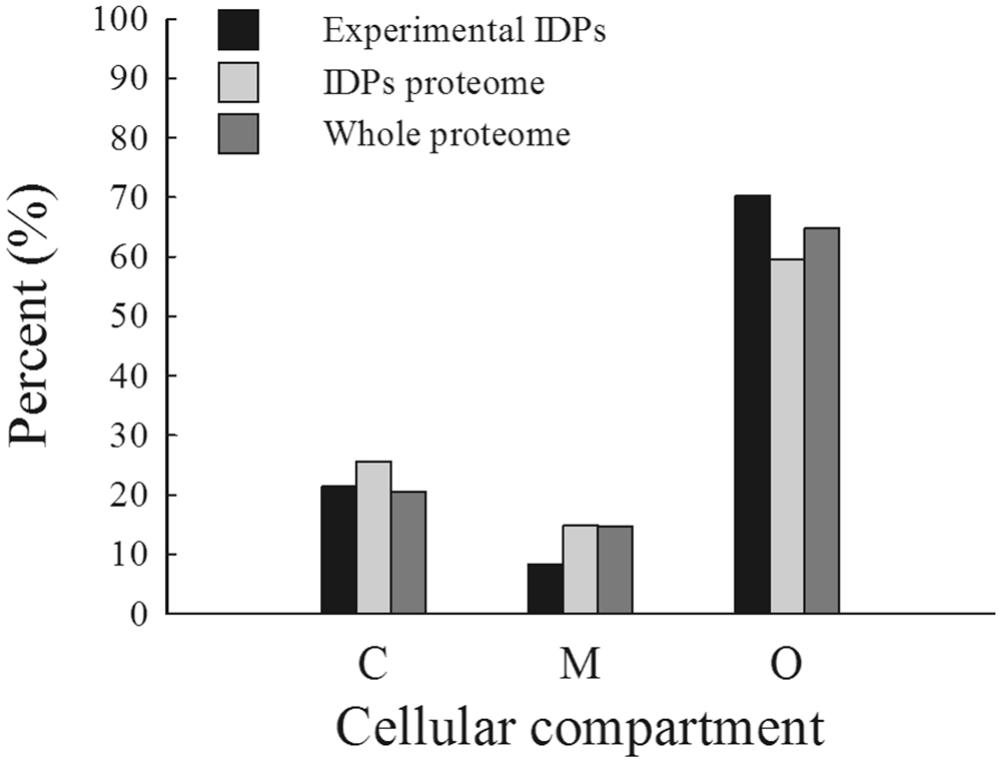
Subcellular localization of IDPs. The distributions of the 299 experimental IDPs (black bars), of the 2152 IDPs from *C*. *reinhardtii* proteome (gray bars) and from the whole algal proteome (9418 proteins since protein partial sequences were removed (see list in Supplementary Table S4, dark gray bars) in the chloroplast (C), in the mitochondrion (M) and in the other compartments (O) are shown. These localizations were predicted using PredAlgo (https://giavapgenomes.ibpc.fr/predalgo/).

Using PredAlgo, we further analyzed the location of (i) the 299 experimental IDPs, (ii) the 2152 IDPs and (iii) the whole *C*. *reinhardtii* proteome (9418 proteins that are listed in Supplementary Table S4) mentioned above (Fig. 4). Among the 299 experimental IDPs, 8.4% proteins were predicted to be addressed to the mitochondrion (M) and 21.4% proteins to the chloroplast (C). The rest (70.2%) was located in other compartments ((O), e.g. the nucleus, the endoplasmic reticulum, etc.) but could not be analyzed any further because there was no predictor available for these compartments. The same trend, less IDPs in the mitochondrion, in the chloroplast and in the other compartments (M < C < O), was followed for all the other predicted IDPs. However, in the mitochondrion, less disordered proteins were found in our experimental dataset compared to the predicted IDPs (14.8%). As regard to the whole proteome the same percent of proteins were also found with 14.7% in the mitochondrion and around 20% in the chloroplast.

### Analysis of amino acid composition

**Figure 5:**
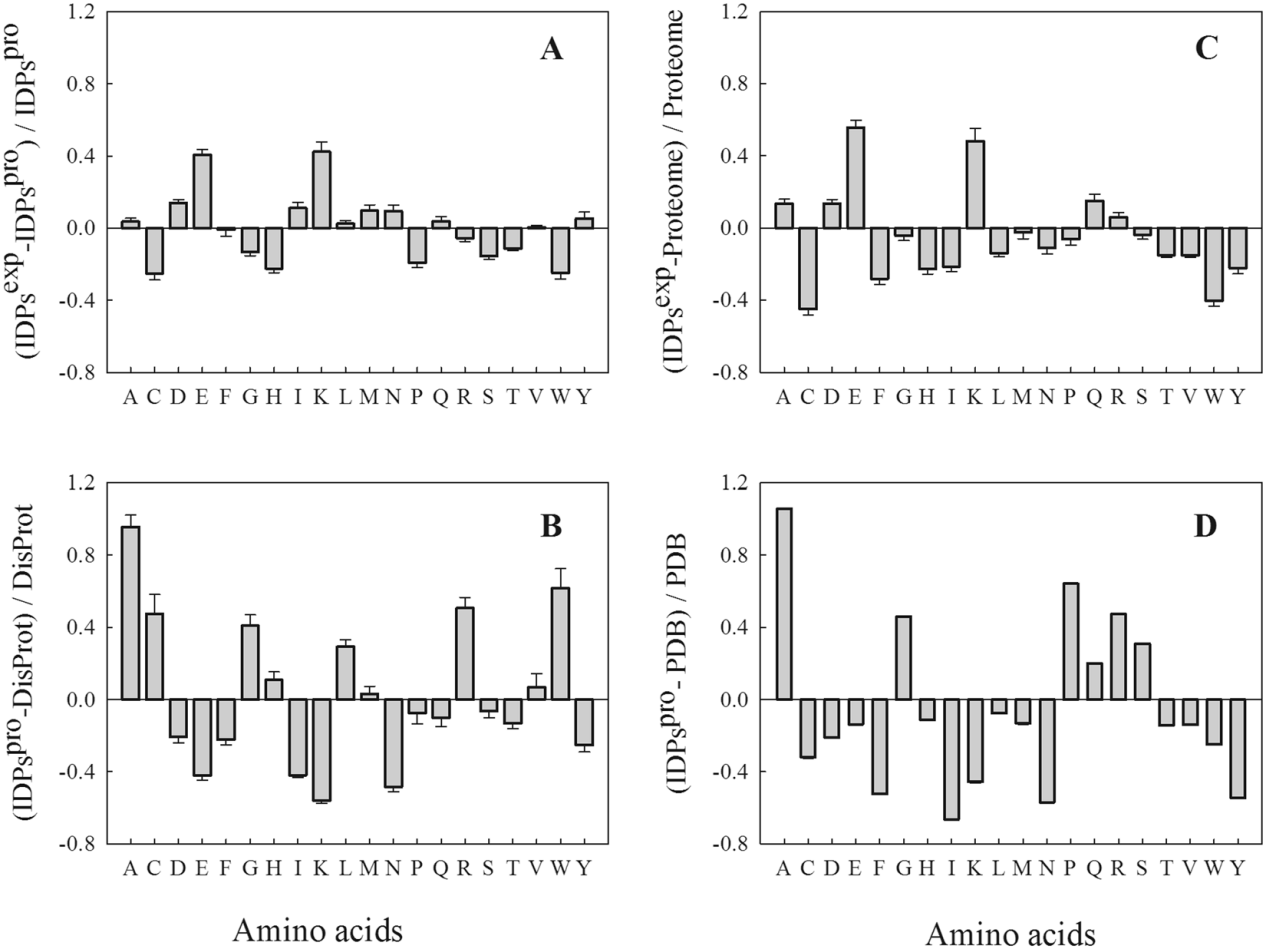
Amino acid composition of IDPs. Amino acid comparison, using Composition profiler (http://www.cprofiler.org/)^82^, of IDPs experimentally (IDPs^exp^) found and the 2152 IDPs (IDPs^Pro^) from *C*. *reinhardtii* (**A**), of the 2152 IDPs from *C*. *reinhardtii* (IDPs^Pro^) and IDPs from DisProt 3.4^24,25^ (**B**), of IDPs experimentally found (IDPs^exp^) and the whole proteome from *C*. *reinhardtii* (**C**), of the 2152 IDPs (IDPs^Pro^) from *C*. *reinhardtii* and of the structured or globular proteins from the protein data bank PDB Select 25^83^ (**D**).

Using Composition profiler, we analyzed the amino acid compositions of the experimental IDPs and compared them to those of (i) the 2152 predicted IDPs, (ii) the IDPs from DisProt 3.4 database, (iii) the whole *C*. *reinhardtii* proteome and (iv) to those of the globular proteins from the Protein Data Bank (PDB Select 25). When experimental IDPs were compared to the 2152 predicted IDPs, a few differences were observed compared to all the other sets. However, some residues such as Glu, and Lys were higher and Cys, His, Pro and Trp were lower in the experimental IDPs (Fig. 5A). The 2152 predicted IDPs from *C*. *reinhardtii vs* classic IDPs^24,25^ had a biased amino acid composition (Fig. 5B). They had a higher content in Ala, Arg, Gly, Leu and unexpectedly, in Trp and Cys residues than classic IDPs, a lower content in Asn, Ile, Glu, and Lys and a similar content in other residues. When the experimental IDPs were compared to the whole proteome (Fig. 5C), as expected they had a higher content in charged residues such as Glu, Lys and lower content in Cys, and all aromatic residues (Phe, Trp and Tyr). The 2152 predicted IDPs compared to globular proteins from the PDB S25 (Fig. 5D), were depleted in Asp, Glu and Lys unexpectedly, but were enriched in structure-breaking residues (Pro and Gly) and in Ala, Arg and Ser residues; as expected, they were also depleted in hydrophobic and aromatic residues (Phe, Trp, Tyr), and had low content of order-promoting amino acid residues (Ile, Met, Leu, Val, Asn, Cys).

### Molecular functions of IDPs

**Figure 6:**
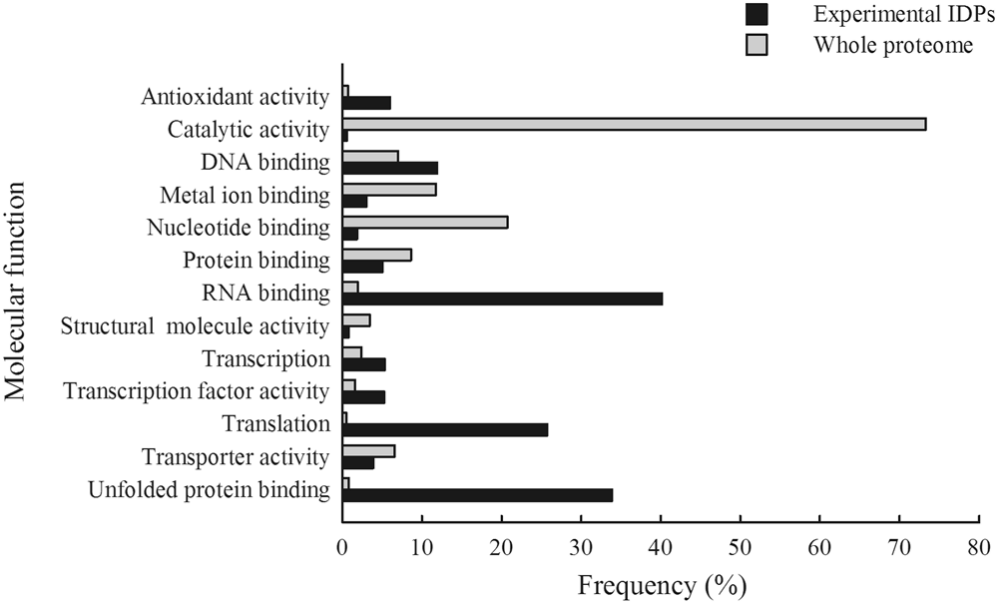
Classification of molecular functions of IDPs and of the proteome from *C*. *reinhardtii*. Classification of the 299 experimental IDPs (black bars) and of the 7058 proteins (gray bars) with known molecular functions (https://genome.jgi.doe.gov/cgi-bin/ToGo?accession = GO:0003674&species = Chlre4) is based on gene ontology (GO) terms.

We analyzed the predicted molecular functions associated with the flexible proteins identified systematically with the four disorder algorithms and classified them using GO terms from the molecular function ontology. We searched for the proteins having the same molecular function in the whole proteome. For the two sets we calculated the frequency of proteins being in the same molecular function category (Fig. 6). The results showed that IDPs were most abundant in the GO terms, RNA binding, unfolded protein binding, translation and DNA binding; they were also more abundant in the GO terms antioxidant activity, transcription and transcription factor activity. Most proteins from the whole proteome could be clustered in the GO term catalytic activity but only few IDPs were present in this category. Proteins clustered in the GO terms, nucleotide binding, metal ion binding, protein binding and transporter activity were slightly more frequent in the whole proteome than in the experimental IDPs.

## Discussion

IDPs contain three times less aggregation prone regions than globular proteins, in particular, they lack a hydrophobic core that can be exposed under denaturing conditions, and remain soluble even at high temperature and under acid treatment^34^. Therefore as previously reported for other organisms^34–36^, we used these treatments to isolate IDPs. In *C*. *reinhardtii*, heat-treatment was a better method to isolate IDPs than acid-treatment and allowed 20 times more IDPs to be recovered in the soluble fraction. This fraction contained 7% of the total protein extract from which we identified 682 soluble proteins called heat-resistant proteins. This set of proteins is probably not an exhaustive list of heat-resistant proteins since some proteins may have precipitated due to aggregation of globular domain surrounded by IDR; others were out of the range of molecular mass investigated in this study and others may have not been found in the LC-MS/MS analysis.

As a result of amino acid bias, sequence complexity, hydrophobicity, charge and other sequence attributes, sites of PTM are frequently associated with IDPs. Phosphorylation was reported to be one of the most common PTMs and over-represented in the flexible regions of eukaryotic proteins, including plants^12,48^. Indeed, we experimentally identified Serine/arginine-rich splicing factors (SR proteins) consistent with what has been shown for other organisms^49,50^. Moreover, we found that 50% of the 682 proteins studied in this work were present in the recently published phosphoproteome of *C*. *reinhardtii*^14,51^ indicating that they can be phosphorylated. It has been shown *in silico* that there is a positive relationship between phosphorylation and content of flexibility in in algae proteome analysis^52^. Combining two non-targeted experimental approaches, the characterization of flexible proteins (this work) and the phosphoproteome by Wang *et al*. of *C*. *reinhardtii*^14^, we were able to list proteins that were both flexible and phosphorylated. Since PTM, especially phosphorylation, and flexibility are two key factors involved in protein-protein interaction and regulation, further research is likely to detect important regulators in *C*. *reinhardtii*. This study will help to develop driven approach to answer more specific biological questions.

The different percent of proteins in the different compartments of the cell reveals that the mitochondrion and the chloroplast contain a lower proportion of IDPs as previously shown^53^ and that other compartments (including the nucleus) contain a higher proportion of IDPs as described for other organisms^10^. The chloroplast and mitochondria are ancient organelles with a prokaryotic origin, which probably explains their low level of disordered elements^54^. In addition, evolutionary pressure might have forced nuclear proteins to acquire disordered regions.

Though the experimental IDPs *vs* all predicted IDPs from *C*. *reinhardtii* were enriched in Glu, Lys and contained less Trp residues, both have common features with classic IDPs^5,8,9,47^ and present some peculiarities. The content in Ala and Gly residues is even higher in IDPs from this green alga than in higher plants^55^. All together these results imply that IDPs have amino acid compositions that are distinct from globular proteins but are also species-specific within the same kingdom, Plantae.

Disorder is less frequent in enzymes and many proteins involved in catalytic activity are structured, and as expected, we found only few IDPs clustered in this category. However, benefitting from their biased amino acid composition and thereby highly conformational flexibility, IDPs can bind multiple partners to perform their particular functions^10,30^. Indeed many IDPs in our study are associated with nucleic acids binding. Moreover, as reported in the literature, IDPs found in plants are associated with many stress-response processes, acting as protein chaperones^56^ or protecting other cellular components^2^. We have identified 20 IDPs related to unfolded protein binding or chaperone-function, with some illustrative examples, Hsp33^57–60^, Hsp70 and Hsp90^61^, belonging to the family of heat-shock proteins. Hsp70 and Hsp90 were also found in the Chlamydomonas phosphoproteome^14^ indicating that they are phosphorylated. Though these chaperones play crucial roles in relation to their flexibility, they are understudied in *C*. *reinhardtii* compared to other organisms^62,63^.

The similarity between the chloroplast ribosome and the 70S bacterial ribosome^64^ is such that a lower content of disorder is expected for the chloroplast ribosomal proteins compared to the cytosolic ones. This is in agreement with our results. We experimentally identified 14 chloroplast, 42 cytosolic and one mitochondrial ribosomal proteins (L29) (Tables 3 and 4). 21 of these ribosomal proteins were confirmed as flexible by the four algorithms, among which were 17 cytosolic ribosomal proteins. Three large chloroplast ribosomal proteins were also confirmed by the four predictors, two (L15 and L34), were found in other plants^53^ while one (L32) was specific to *C*. *reinhardtii* and of interest, was present in the phosphoproteome. On the contrary, other chloroplast ribosomal proteins were not confirmed as IDPs by all the predictors but have been described as flexible in higher plants such as S5, S21, L11, L18 and L24^53^ (Table 3). This suggests that flexibility in ribosomal proteins is probably under estimated when the four predictors are taken into account. By homology with the *E*. *coli* 70S ribosome, it is expected that the L7/L12 stalk of the chloroplast ribosome remains flexible^65^ and it was experimentally confirmed. As expected, we also identified the core histones (H2A, H2B, H3, H4) and the linker histone (H1 family) as IDPs^66–68^.

**Table 3:** Experimental disordered proteins within the chloroplast ribosome in *C*. *reinhardtii*. Accession numbers are from Phytozome v12.1 and from UniProt. NF stands for accession number not found. P, D, I and F corresponds to PONDR, DisEMBL, IUPred and FoldIndex, respectively.

Accession	Accession	Name	P	D	I	F
Cre16.g659950.t1.1	A8J8M5	S5	+	−	+	+
Cre12.g494450.t1.2	A8JDN8	S16	+	−	−	+
NF	NP_958370	S19	+	+	+	+
Cre12.g494750.t1.2	A8JDN4	S20	+	+	−	+
Cre01.g017300.t1.2	A8HPN4	S21	+	−	−	+
Cre13.g581650.t1.2	A8HTY0	L7/L12	+	+	−	−
Cre10.g423650.t1.2	A8ICE4	L11	+	−	−	−
Cre14.g612450.t1.2	A8JAL6	L15	+	+	+	+
Cre01.g052100.t1.2	A8HNJ8	L18	+	−	−	+
Cre16.g652550.t1.2	A8J9D9	L24	+	−	−	+
Cre07.g352850.t1.2	A8IUC3	L32	+	+	+	+
Cre01.g030050.t1.2	A8HQG3	L34	+	+	+	+
Cre02.g083950.t1.1	A8I8A3	P3	+	+	−	+
Cre12.g519180.t4.1	A8J641	P7	+	−	+	+

**Table 4:** Experimental disordered proteins within the cytosolic ribosome in *C*. *reinhardtii*. P, D, I and F corresponds to PONDR, DisEMBL, IUPred and FoldIndex, respectively. Accession numbers were from Phytozome v12.1 and from UniProt. NF stands for accession number not found.

Accession	Accession	Name	P	D	I	F	Accession	Accession	Name	P	D	I	F
Cre02.g102250.t1.2	A8I4P5	S3	+	−	−	−	Cre12.g528750.t1.2	A8J597	L12	+	−	−	+
Cre13.g568650.t1.2	A8HS48	S3a	+	+	−	+	Cre14.g630100.t1.2	A8IUV7	L13	+	+	+	+
Cre09.g400650.t1.2	A8J1G8	S6	+	+	+	+	Cre12.g532550.t1.1	A8IVU0	L13a	+	−	−	+
Cre11.g467578.t1.1	A8JF05	S8	+	+	+	+	Cre17.g701200.t2.1	A8IQE3	L14	+	+	−	+
Cre04.g214503.t1.1	A8J9T0	S12	+	−	−	−	Cre12.g512600.t1.2	A8IKZ2	L18	+	+	−	+
Cre07.g331900.t1.2	A8IGY1	S13	+	+	−	+	Cre02.g075700.t1.2	A8IA18	L19	+	+	+	+
Cre12.g498250.t1.2	A8JGK1	S17	+	+	+	+	Cre06.g278135.t1.1	A8J951	L21	+	+	+	+
Cre16.g660150.t1.2	A8J8M9	S20	+	+	−	+	Cre07.g357850.t1.2	A8JI94	L22	+	+	−	+
Cre03.g203450.t1.2	A8IXG3	S21	+	−	−	−	Cre09.g391097.t2.1	A8J1A3	L24	+	+	+	+
Cre10.g456200.t1.2	A8I0I1	S24	+	+	+	+	Cre01.g040000.t1.2	A8HMG7	L26	+	+	+	+
Cre08.g382500.t1.2	A8IZ36	S25	+	+	+	+	Cre11.g467578.t1.1	A8JF05	L28	+	+	+	+
Cre06.g273600.t1.2	A8HVK4	S27a	+	+	+	+	NF	A8ILG8	L31	+	+	−	+
Cre12.g510450.t1.2	A8IKP1	S28	+	−	−	+	Cre14.g617900.t1.2	A8HNX3	L35	+	−	−	+
Cre08.g358556.t1.1	A8JIE5	S29	−	−	−	+	Cre12.g484050.t1.2	A8JHU2	L36	+	−	+	+
Cre16.g666301.t1.2	A8JF66	S30	+	+	+	+	Cre06.g310700.t1.2	A8IM74	L36a	+	−	−	+
Cre14.g621450.t1.2	A8HP55	L5	+	+	−	+	Cre10.g430400.t1.2	A8IBG1	L37	+	+	+	+
Cre01.g011000.t1.2	A8HP90	L6	+	+	−	+	Cre06.g257150.t1.2	A8HY08	L37a	+	−	−	+
Cre12.g529651.t1.1	A8J567	L7a	+	+	−	+	Cre07.g325746.t1.1	Q8GUQ9	L38	+	−	−	+
Cre12.g535851.t1.1	A8IVK1	L8	+	+	+	+	NF	A8J1Q3	L39	+	+	+	+
NF	P50884	L12	+	−	−	+	Cre01.g007051.t1.2	A8JCX9	L40	+	+	−	+
Cre12.g520500.t1.1	A8J5Z0	P0	+	+	−	+	Cre02.g143050.t1.2	A8J0R4	P2	+	+	+	−

Thirteen flagellar associated proteins over the 29 predicted to be highly flexible, were found in the phosphoproteome, in agreement with previous reports showing that phosphorylation is a key modification involved in flagellar assembly/disassembly in *C*. *reinhardtii*^69,70^. We also found 27 disordered proteins that play a very critical role in cytoskeleton assembly.

IDPs in *C*. *reinhardtii* may provide a fast and efficient mechanism to respond to changing environmental conditions and therefore play very important roles as described in other organisms^53^. Indeed, we also found many IDPs involved in the regulation of translation and transcription.

To conclude, although disorder is emerging to have numerous important functions in a cell, in plants it has been largely understudied, and the work reported here is the first large-scale experimental investigation of the intrinsically disordered proteome in *C*. *reinhardtii*. Indeed, few IDPs have been biochemically characterized in *C*. *reinhardtii*, CP12^43,71–73^, EPYC1^46^ and an IDR-containing protein, adenylate kinase 3^74^. In this work, a central pipeline for the extraction, identification, characterization and analysis of IDPs was developed. Taken together, our experimental results and bioinformatic analysis lead to a greater knowledge of IDPs and show that structural flexibility is widespread, and likely important, in many biological processes in *C*. *reinhardtii*.

## Methods

### Protein extraction

*C*. *reinhardtii* was grown mixotrophically in Tris-acetate-phosphate (TAP) medium at 25 °C with vigorous shaking 90 rpm under 50 µmol photon m^−2^ s^−1^ photosynthetically active radiation^75^. Cultures (50 mL, 5 replicates) from the exponential phase were centrifuged at 4,000 g for 15 min at 4 °C (Beckman Coulter Allegra® X-15R Centrifuge (Pasadena, CA, USA); rotor: 4750 A), then stored at −80 °C. Cells were broken by sonication in lysis buffer (15 mM Tris, 0.1 mM EDTA at pH 7.5) supplemented by protease inhibitors 40 µg mL^−1^ (Sigma-Aldrich, P2714); the homogenate was centrifuged at 11,000 g, 4 °C for 30 min (2–16KC centrifuge using a 12132-H rotor, Sigma-Aldrich, Saint-Louis, MO, USA) to isolate the supernatant that mainly contained non-membrane proteins.

### Acid and heat treatments

Acid-resistant proteins were extracted by treating with 10% perchloric acid (PCA) or 5% trichloroacetic acid (TCA), on ice for 15 min. The heat-resistant proteins were extracted by boiling the samples at 98 °C for 5 min, 30 min and 1 h. After cooling to room temperature, the insoluble fractions were removed by centrifugation at 11,000 g, 4 °C for 30 min (2–16KC centrifuge using a 12132-H rotor, Sigma-Aldrich, Saint-Louis, MO, USA) and thus, only the soluble extracts containing heat-resistant or acid-resistant proteins were kept and analyzed further^35^. Protein concentration was determined, using the Bio-Rad reagent protein assay (Bio-Rad Laboratories, Hercules, CA, USA) with bovine serum albumin as a standard.

### Sodium dodecyl sulphate (SDS) polyacrylamide gel electrophoresis (PAGE) of soluble proteins

Protein migration was performed on 12% polyacrylamide gel Mini-PROTEAN^®^ Tetra Cell (Biorad, Hercules, USA). Extracts were incubated for 5 min at 94 °C with 10% SDS, 10 mM DTT, 20% glycerol, 0.2 M Tris-HCl pH 6.8 and 0.05% Bromophenol Blue and 10 µg of each heated-sample were loaded onto the gels. After running, the gels were stained with Coomassie Brilliant Blue R250.

### Identification of proteins by mass spectrometry

The most intense bands separated by SDS-PAGE were cut and submitted to trypsin digestion as previously described^76^. LC-MS/MS analyses were performed on an ESI-Q-Exactive plus mass spectrometer (ThermoFisher) coupled to a nano liquid chromatography (Ultimate 3000, Dionex). Solubilized tryptic peptides in 0.05% (v/v) TFA/2% (v/v) acetonitrile were loaded onto a nano trap (Acclaim PepMap100, 100 µm × 2 cm, 5 µm, 100 Å, Dionex) before elution onto a C18 column (Acclaim PepMap RSLC, 75 µm × 150 mm, 2 µm, 100 Å, Dionex). A linear gradient from 6% to 40% of mobile phase B (0.1% (v/v) formic acid (FA)/80% (v/v) acetonitrile) in mobile phase A (0.1% (v/v) FA) was applied for 52 min. The peptides were detected into the mass spectrometer in a positive ion mode, using a Top 10 Data Dependent workflow with a 60 s dynamic exclusion. One scan event full MS in the Orbitrap at 70 000, in a 350–1900 m/z range, was followed by a fragmentation MS/MS step, at 17 500, of the 10 top ions, in the Higher Energy Collisional Dissociation cell set at 27.

For protein identification, spectra were processed by the Proteome Discoverer software (Thermo Fisher Scientific, versions: 1.4.0.288 and 2.1.0.81) using the Sequest HT algorithm including the Protein Center annotation aspects (biological process, cellular component, molecular function).

To identify the heat-resistant proteins, the search was performed using *C*. *reinhardtii* databank (Taxonomy ID 3055, 15313 sequence entries) downloaded from the non-redundant NCBI databank and/or from Phytozome v12.1 (19526 sequence entries). The following parameters were set: enzyme: trypsin; dynamic modifications: oxidation/+ 15.995 Da (M), phosphorylation/+ 79.966 Da (Y, S, T), static modification: carbamidomethyl/+ 57.021 Da (C); mass values: monoisotopic; precursor mass tolerance: ± 10 ppm; fragment mass tolerance: ± 0.02 Da; missed cleavages: 2. Proteins were considered as identified by 2 unique “rank 1” peptides, as shown by the two best Peptide Spectrum Matches (PSM), passing the high confidence filter, with validation on q-Value (Strict Target FDR: 0.01) and maximum Delta Cn: 0.05.

### Computational evaluation of disorder

We selected proteins with PSM higher than two and removed proteins where only partial sequence was available. We used four different algorithms^47^: (i) PONDR VL-XT (http://www.pondr.com/cgi-bin/PONDR/pondr.cgi) that is based on artificial neural networks, using a variety of physiochemical properties of the input protein chain including amino acid compositions, aromaticity, flexibility, hydropathy, and hydrophobicity^77^; (ii) FoldIndex (http://bip.weizmann.ac.il/fldbin/findex) that is based on the average residue hydrophobicity and net charge of the sequence^78^; (iii) DisEMBL Remark-465 (http://dis.embl.de/) that is also a method based on artificial neural networks trained for predicting several definitions of disorder, in particular, it is trained on evolutionarily conserved sequence features of disordered regions that have missing residues in high-resolution X-ray structures; (iv) IUPred (http://iupred.enzim.hu/) that predicts intrinsic disorder based solely on propensities/properties of amino acids of the input protein sequences^79^. In our study we therefore used these different types of algorithms to increase reliability of our analysis.

### Cellular compartment

To predict where the proteins were targeted (mitochondrion, chloroplast, and other compartments), PredAlgo (https://giavap-genomes.ibpc.fr), the most reliable software for Chlamydomonas and related green algae species (Chlorophyta), was used^80^.

### Amino acid residues comparison

The comparison of the amino acid composition of the different sets of proteins from *C*. *reinhardtii* was analyzed by Composition profiler (http://www.cprofiler.org/)^24,25^.

### Functional classification

The molecular function of each disordered protein was attributed according to the Gene Ontology (http://geneontology.org/) and JGI Genome Portal (http://genome.jgi.doe.gov/)^54,81^.

## Electronic supplementary material

Supplemental Data Fig S1 Tables S2 and S3

List of the 682 heat-resistant proteins experimentally identified as potential IDPs, from Chlamydomonas reinhardtii.

List of the C. reinhardtii proteins (full length) and their cellular compartment predicted by PredAlgo.
