# Author Correction: Exploring intrinsically disordered proteins in *Chlamydomonas reinhardtii*

**DOI:** 10.1038/s41598-018-31276-x

**Published:** 2018-08-29

**Authors:** Yizhi Zhang, Hélène Launay, Antoine Schramm, Régine Lebrun, Brigitte Gontero

**Affiliations:** 1 Aix Marseille Univ, CNRS, BIP, UMR 7281, IMM, 31 Chemin J. Aiguier, 13402 Marseille, Cedex 20 France; 2 Aix Marseille Univ, CNRS, AFMB, UMR 7257 Marseille, France; 3 Plate-forme Protéomique, Marseille Protéomique (MaP), IBiSA labeled, IMM, FR 3479, CNRS, B.P. 71, 13402 Marseille, Cedex 20 France

Correction to: *Scientific Reports* 10.1038/s41598-018-24772-7, published online 01 May 2018

The original version of this Article contained errors in the spelling of the authors Yizhi Zhang, Hélène Launay, Antoine Schramm, Régine Lebrun & Brigitte Gontero which were incorrectly given as Zhang Yizhi, Launay Hélène, Schramm Antoine, Lebrun Régine & Gontero Brigitte respectively.

These errors have now been corrected in the PDF and HTML versions of the Article, and in the accompanying Supplementary Data file.

