# Supplementary material for: Exploring intrinsically disordered proteins in Chlamydomonas reinhardtii
Source: Sci Rep. 2018 May 1;8:6805. doi: 10.1038/s41598-018-24772-7 (PMC5931566; doi:10.1038/s41598-018-24772-7)
Supplement: Supplementary file 1 — Supplemental Data Fig S1 Tables S2 and S3 [file 41598_2018_24772_MOESM1_ESM.pdf]

Supplemental Data from the original manuscript by Zhang *et al*. entitled

Exploring intrinsically disordered proteins in *Chlamydomonas reinhardtii*

Yizhi Zhang, Hélène Launay, Antoine Schramm, Régine Lebrun, Brigitte Gontero

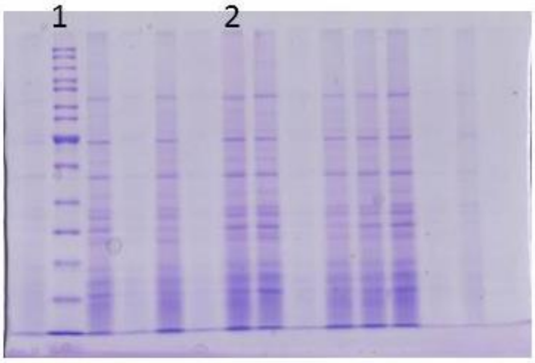

Figure S1. **SDS-PAGE.** Molecular weight markers (Euromedex. unstained protein ladder, (Lane 1). 10 µg of non-membrane heat-treated proteins from *C. reinhardtii* were loaded and separated on 12% polyacrylamide gels under denaturing conditions and stained with Coomassie Blue. Bands from Lane 2 were cut and further analyzed while the other lanes were replicates but were not used in this study.

Supplementary Table S2. List of phosphopeptides identified by mass spectrometry. 19 proteins were found as disordered proteins (see % of disorder predicted by PONDR) and phosphorylated in our study. The phosphorylated residues in the phosphopeptides identified by mass spectrometry are in bold and indicated by an asterisk. AAs represents the number of amino acid residues. Grey colour showed the proteins that were absent in the phosphoproteome recently published by Wang *et al*, 2014.*

NCBI Accession	Name	AAs	%disorder	Phosphopeptide identified by MS
XP_001698601	Predicted protein	2076	74.2	AEAAEPAGPAAVEEEAP**T*S**PR
XP_001696302	Predicted protein	720	79.4	**T* S***SSGGTAGAALTAPSAPR
XP_001698509	Flagellar associated protein	515	74.4	SEAVG **S***T SVK **S***AK
XP_001697581	Hypothetical protein	458	89.5	QLGG **S***GQQLSGLGGASR
XP_001703197	Flagellar associated protein	443	75.2	GG **S***GSGAQAAAAPTGQSFSFGAKPEAK
XP_001695654	Predicted protein	413	85.7	HMQEVHGVSIQHD **S***DDER
XP_001702497	Predicted protein	104	62.5	RPA **S***PPNPQAVLQAK
XP_001695463	Predicted protein	494	74.5	ATAD **S***DDEGESAKPASTSLGAAAAASGK
				SVGGAPD **S***PQLPGGGAAGPSPEWDDSPAR
XP_001691264	GTPase activation protein	1138	65.7	VPEPGASAPK **S***PLATPVEAPAQAASSPEAEVK
XP_001697455	Eukaryotic initiation factor	197	64	ASLEDMMPPSD **S***EESDQEEGQAGASAAAVAGAAK
A2T2X4	Intraflagellar transport protein 46	344	45.5	TL **S***PTGYEAGK
XP_001695456	CC domain 6-like protein	335	65.4	SP **S***STNNSFDR
XP_001698782	Hypothetical protein	577	83.5	QSALQQQLAQVQVLALQQE**T***AAR
XP_001690066	Telomere binding protein 1	221	47.1	T **S***WQDLKDK
CAC94941	Putative blue light receptor	749	37.3	TIVDDV**T***IAVEK
XP_001699840	Eukaryotic initiation factor	165	47.3	EYTVIDITDEGIVSMMDESGNTRDDMFLPTG**T***DESDKLAEIMK
XP_001700625	Predicted protein	168	61.9	SPA **S***DTYIIFGEAK
				**S***KNILFVIQSPDVFK
				NILFVIQ **S***PDVFK
XP_001694699	O_2-evolving enhancer protein1	291	33.3	LTYTLDAMSG **S***FK

XP_001695560	Predicted protein	145	26.9	ALGK**S***PTEAEIK

*Wang, H. *et al*. The global phosphoproteome of *Chlamydomonas reinhardtii* reveals complex organellar phosphorylation in the flagella and thylakoid membrane. *Mol Cell Proteomics*, **13**, 2337-2353 (2014).

Supplementary Table S3. List of proteins found both in our study and in the *C. reinhardtii* phosphoproteome (Wang, H. *et al*. The global phosphoproteome of *Chlamydomonas reinhardtii* reveals complex organellar phosphorylation in the flagella and thylakoid membrane. *Mol Cell Proteomics*, **13**, 2337-2353 (2014)).

**Uniprot Accession**	**NCBI Accession**	**Name**
A2T2X4	A2T2X4	Intraflagellar transport protein 46
Q945T1	AAK96224	Co-chaperone CGE1 precursor isoform b
Q2HZ24	ABC88602	Putative ferredoxin
A8HPN4	XP_001689480	Plastid ribosomal protein S21
A8HPT6	XP_001689504	Predicted protein
Q6RCE1	XP_001689563	Intraflagellar transport protein 74/72
A8HQH6	XP_001689584	Predicted protein
A8HQM6	XP_001689600	Predicted protein
A8HQT1	XP_001689618	Protein disulfide isomerase
A8HMD3	XP_001689665	Hypothetical protein
A8HMG7	XP_001689675	Ribosomal protein L26
A8HMQ1	XP_001689702	Aconitate hydratase
A8HN94	XP_001689751	Predicted protein
Q84XR9	XP_001689786	Thioredoxin x
A8HNX3	XP_001689832	Ribosomal protein L35
A8HP02	XP_001689841	Predicted protein
A8HP06	XP_001689842	Succinate dehydrogenase subunit A
A8HP55	XP_001689859	Ribosomal protein L5

A8HP84	XP_001689871	Glyceraldehyde-3-phosphate dehydrogenase
A8HP90	XP_001689873	Ribosomal protein L6
A8HPE9	XP_001689890	Predicted protein
A8HPM8	XP_001689925	Predicted protein
Q39568	XP_001690066	G-strand telomere binding protein 1
A8HMA8	XP_001690119	Acetyl-coa biotin carboxyl carrier
A8HMJ6	XP_001690151	Predicted protein
A8HMS5	XP_001690177	Predicted protein
A8HMW1	XP_001690192	Predicted protein
A8HNG8	XP_001690252	Uncharacterized lumenal polypeptide
A8HNY3	XP_001690288	Predicted protein
A8IHY1	XP_001690324	Hypothetical protein
A8IIC7	XP_001690351	Hypothetical protein
A8IIP7	XP_001690375	26S proteasome regulatory subunit
Q546J6	XP_001690415	SF-assemblin
A8IGY1	XP_001690424	Ribosomal protein S13
A8IHF8	XP_001690456	NimA-related protein kinase 8
Q6UPR1	XP_001690509	NimA-related protein kinase 5
A8IGH1	XP_001690591	Superoxide dismutase [Fe]
A8IH77	XP_001690629	Subunit H of photosystem I
A8IJQ1	XP_001690805	Iron-sulfur cluster assembly protein
A8IK44	XP_001690833	Predicted protein
A8IJ27	XP_001690957	Predicted protein
A8IJ76	XP_001690963	Hypothetical protein
A8ILB6	XP_001691099	UPF3 protein
A8IMV0	XP_001691227	Hypothetical protein
A8IMZ5	XP_001691232	Magnesium chelatase subunit I

A8INH1	XP_001691264	Potential GTPase activation protein
Q2HZ24	XP_001691381	Apoferredoxin
A8IP08	XP_001691449	Predicted protein
A8IP53	XP_001691459	Translation initiation protein
A8IPB8	XP_001691464	Predicted protein
A8IAW5	XP_001691500	Hypothetical protein
A8IR30	XP_001691526	Iron-sulfur cluster assembly protein
A8IRA4	XP_001691553	Protein kinase
A8IQU3	XP_001691632	Beta subunit of mitochondrial ATP synthase
A8IRB7	XP_001691710	Predicted protein
A8IRH4	XP_001691826	Metacaspase type II
A8IRV0	XP_001691869	HSP70-HSP90 organizing protein
A8IRV6	XP_001691870	Eukaryotic initiation factor
Q540H1	XP_001691876	Tubulin alpha-1 chain
A8IS75	XP_001691919	Hypothetical protein
A8IRK5	XP_001691998	Hypothetical protein
A8IRQ1	XP_001692016	Ribose-5-phosphate isomerase
A8IRU6	XP_001692034	Peptidyl-prolyl cis-trans isomerase, cyclophilin-type
A8ISK5	XP_001692126	Hypothetical protein
A8ISS9	XP_001692268	Hypothetical protein
A8ITH3	XP_001692382	Predicted protein
A8ITL0	XP_001692395	Mitochondrial F1F0 ATP synthase associated 60.6 kDa protein
Q945T2	XP_001692412	GrpE nucleotide release factor
A8IU13	XP_001692457	Predicted protein
A8IUB8	XP_001692499	Predicted protein
A8ITH8	XP_001692504	Chaperonin 60B2
A8ITQ1	XP_001692527	P23 co-chaperone of HSP90 system

A8IU79	XP_001692603	WW domain protein
A8IUC3	XP_001692618	Plastid ribosomal protein L32
A8IUV6	XP_001692669	Predicted protein
A8IUG8	XP_001692826	Plastidic thioredoxin-like protein
A8IW20	XP_001693051	Eukaryotic initiation factor
P93106	XP_001693118	Malate dehydrogenase
A8IXT4	XP_001693239	Hypothetical protein
A8IWG9	XP_001693253	Predicted protein
A8IWK2	XP_001693262	Ferredoxin thioredoxin reductase, catalytic chain
A8IXU7	XP_001693387	Phototropin
A8HRZ0	XP_001693443	Histone H1
A8HSE5	XP_001693473	Predicted protein
A8HTX7	XP_001693576	Mitochondrial F1F0 ATP synthase associated 31.2 kDa protein
A8HUK0	XP_001693615	Peptidyl-prolyl cis-trans isomerase, FKBP-type
A8HUN2	XP_001693621	SR protein factor
A2PZC2	XP_001693685	UDP-Glucose:protein transglucosylase
A8HS48	XP_001693710	Ribosomal protein S3a
Q4U0V9	XP_001693726	CTR type copper ion transporter
A8HSU1	XP_001693753	Predicted protein
Q6UKY5	XP_001693782	Acyl-carrier protein
A8HTE8	XP_001693784	Qb-SNARE protein, VTI1-family
A8HTG7	XP_001693788	Glycine-rich protein
Q66YD0	XP_001693830	Vesicle inducing protein in plastids 1
A8IXZ0	XP_001693997	Beta tubulin 2
A8IYP4	XP_001694038	Phosphoribulokinase
A8IY98	XP_001694099	Predicted protein
A8IYH9	XP_001694126	Oxygen-evolving enhancer protein 2 of photosystem II

A8IYJ6	XP_001694135	Predicted protein
A8IYP5	XP_001694158	Thioredoxin h2
A8IZ90	XP_001694245	Hypothetical protein
A6Q0K5	XP_001694345	Small protein associating with GAPDH and PRK (CP12)
A8IZS5	XP_001694459	Glycine-rich RNA-binding protein
A8IZX7	XP_001694490	Flagellar associated protein
A8IZQ8	XP_001694569	Predicted protein
A8IZV5	XP_001694594	Bifunctional GTP cyclohydrolase II
A8J087	XP_001694669	Vasa intronic gene
A8J098	XP_001694675	Flagellar associated protein
A8J0E4	XP_001694699	Oxygen-evolving enhancer protein 1 of photosystem II
Q9FYV4	XP_001694742	Copper chaperone
A8J0D9	XP_001694780	Flagellar associated protein
A8J0R4	XP_001694856	Acidic ribosomal protein P2
A8J152	XP_001694924	Predicted protein
A8J1A3	XP_001694951	Ribosomal protein L24
A8J0N4	XP_001694962	Protein phosphatase 2C
A8J0Q8	XP_001694975	Thioredoxin-related protein CITRX
A8J1G8	XP_001695102	Ribosomal protein S6
A8J1T4	XP_001695163	Dihydrolipoyl dehydrogenase
A8J1V5	XP_001695174	Dihydrolipoamide acetyltransferase
A8J1C2	XP_001695181	Predicted protein
A8J1I6	XP_001695217	Predicted protein
A8J1T5	XP_001695261	Flagella associated protein
A8J237	XP_001695329	Component of the ESCRT-III complex
A8J2J7	XP_001695406	Radial spoke protein 3
A8J246	XP_001695456	Coiled-coil domain 6-like protein

A8J257	XP_001695461	Predicted protein
A8J265	XP_001695463	Predicted protein
A8J2H2	XP_001695521	Predicted protein
A8J2K0	XP_001695533	Predicted protein
A8J2K4	XP_001695535	Predicted protein
A8J2Q0	XP_001695560	Hypothetical protein
A8J354	XP_001695636	Hypothetical protein
A8J2L0	XP_001695654	Predicted protein
A8J2L7	XP_001695659	U6 small ribonucleoprotein F
A8J2L8	XP_001695660	Hypothetical protein
A8J311	XP_001695725	Hypothetical protein
A8J383	XP_001695755	Hypothetical protein
A8J3A8	XP_001695783	Ubiquitin-like protein
A8J3E3	XP_001695908	Peptidyl-prolyl cis-trans isomerase, parvulin-type
A8J3E8	XP_001695911	Hypothetical protein
A8J3U1	XP_001695981	Flagellar associated protein, protease inhibitor-like protein
A8J3V2	XP_001695986	Hypothetical protein
A8J4C9	XP_001696012	Flagellar associated protein
A8J4E7	XP_001696019	Hypothetical protein
A8J4E5	XP_001696101	Hypothetical protein
Q39576	XP_001696172	Histone H1
A8HVK4	XP_001696195	Ribosomal protein S27a
A8HVQ1	XP_001696201	Ribosomal protein S8
A8HW77	XP_001696233	Hypothetical protein
A8HWA8	XP_001696238	Pyridine nucleotide binding protein
A8HWC5	XP_001696241	Hypothetical protein
A8HX54	XP_001696302	Predicted protein

A8HXN9	XP_001696339	Subunit of GARP complex
A8HYR6	XP_001696422	Subunit of the circadian RNA-binding protein CHLAMY 1
A8HYV3	XP_001696432	Heat shock protein 70B
A8HVV6	XP_001696483	Hypothetical protein
A8HX38	XP_001696568	Eukaryotic translation elongation factor 1 alpha 1
Q7X7A7	XP_001696613	14-3-3 protein
A8HY43	XP_001696624	Thylakoid lumenal protein
A8HYD5	XP_001696637	Glycine cleavage system, H-protein
A8HYF5	XP_001696639	Predicted protein
A8HYK1	XP_001696645	Flagellar associated protein
A8HYP5	XP_001696653	Predicted protein
A8HYU5	XP_001696661	S-Adenosylmethionine synthetase
A8J4I5	XP_001696675	Hypothetical protein
A8J4M0	XP_001696688	Hypothetical protein
A8J4Z4	XP_001696742	Mitochondrial F1F0 ATP synthase associated 45.5 kDa protein
A8J513	XP_001696755	Nucleosome assembly protein
A8J537	XP_001696763	Catalase/peroxidase
A8J4I2	XP_001696769	SR protein factor
A8J4N7	XP_001696785	Protein phosphatase 2C
A8J4Q7	XP_001696792	Rieske [2Fe-2S] protein
A8J5K6	XP_001696896	Hypothetical protein
A8J601	XP_001696929	Paf1 complex component
A8J597	XP_001696972	Ribosomal protein L12
A8J5K4	XP_001697006	Nucleolar protein, component of C/D snoRNPs
A8J5N1	XP_001697015	Hypothetical protein
A8J5S4	XP_001697033	Hypothetical protein
A8J696	XP_001697082	Hypothetical protein

A8J6C3	XP_001697099	Phosphoglucan water dikinase
A8J6L2	XP_001697145	Deflagellation inducible protein
A8J6P6	XP_001697253	Hypothetical protein
A8J785	XP_001697332	CF0 ATP synthase subunit II precursor
A8J728	XP_001697365	Hypothetical protein
A8J7H0	XP_001697517	Predicted protein
A8J7H2	XP_001697518	Predicted protein
A8J7J4	XP_001697529	Predicted protein
A8J7P4	XP_001697556	Predicted protein
A8J7W0	XP_001697581	Hypothetical protein
A7UCH9	XP_001697606	Carbonic anhydrase 8
A8J7T7	XP_001697622	Cysteine endopeptidase
A8J814	XP_001697667	Prefoldin-related KE2-like protein
A8J8B3	XP_001697794	Diaminopimelate epimerase
A8J8I8	XP_001697840	Flagellar associated protein
A8J8K9	XP_001697851	Hypothetical protein
A8J8M5	XP_001697858	Plastid ribosomal protein S5
A8J8Q1	XP_001697869	Histone H3 variant
A8J8H5	XP_001697903	Predicted protein
A8J8I6	XP_001697908	VID72-domain protein
A8J8X1	XP_001697985	Hypothetical protein
A8J8X6	XP_001697986	Predicted protein
A8J906	XP_001697999	Hypothetical protein
A8J929	XP_001698012	Hypothetical protein
A8J8V6	XP_001698051	Hypothetical protein
A8J920	XP_001698086	Hypothetical protein
A8J9D9	XP_001698149	Plastid ribosomal protein L24

A8J9G0	XP_001698156	Hypothetical protein
A8J995	XP_001698202	Predicted thylakoid lumen protein
A8J9E8	XP_001698230	Hypothetical protein
A8J9H8	XP_001698246	Flagellar associated protein, nucleoside diphosphate kinase-like protein
A8HZF9	XP_001698307	Hypothetical protein
A8I0K6	XP_001698374	Predicted protein
A8I0Z4	XP_001698396	Nucleolar GTP-binding protein
A8I1R3	XP_001698453	Hypothetical protein
A8HZT8	XP_001698509	Flagellar associated protein
A8I1B8	XP_001698607	RNA export factor
A8J9T0	XP_001698669	Ribosomal protein S12
A8J9R3	XP_001698696	Flagellar associated protein
A8J9X1	XP_001698736	Mitochondrial F1F0 ATP synthase, delta subunit
A8JAH0	XP_001698907	Hypothetical protein
A8JAH1	XP_001698908	Reticulon-like protein
A8JA83	XP_001698921	Dicer-like protein
A8JAL6	XP_001698978	Plastid ribosomal protein L15
A8JAQ8	XP_001699051	Hypothetical protein
A8JAU7	XP_001699067	Hypothetical protein
A8JAV1	XP_001699068	Actin
A8JAT7	XP_001699108	Nuclear SR-like RNA binding protein
A8JBM7	XP_001699330	Hypothetical protein
A8JBF2	XP_001699339	Organellar elongation factor P
A8JBL9	XP_001699367	Hypothetical protein
A8JC40	XP_001699499	Centrin
A8JC42	XP_001699500	Transmembrane ATPase
A8JC04	XP_001699523	Phosphoglycerate kinase

A8JC79	XP_001699601	Flagella associated protein
A8JCA2	XP_001699614	Hypothetical protein
Q96550	XP_001699641	Mitochondrial F1F0 ATP synthase, alpha subunit
A8I2N3	XP_001699643	Flagellar associated protein
A8I2V3	XP_001699660	2-cys peroxiredoxin
A8I3Q0	XP_001699725	Flagellar associated protein, transcriptional coactivator-like protein
A8I439	XP_001699752	Flagellar associated protein
A8I459	XP_001699756	Acetylornithine deacetylase
A8I4P5	XP_001699797	Ribosomal protein S3
A8I297	XP_001699840	Eukaryotic initiation factor
Q9FED4	XP_001699846	Regulator of CO2-responsive genes
A8I2V7	XP_001699891	Hypothetical protein
A8I2E0	XP_001700058	Superoxide dismutase [Mn]
Q6V504	XP_001700114	NADH : ubiquinone oxidoreductase 12.5 kDa subunit
A8JCS8	XP_001700192	Predicted protein
A8JCP3	XP_001700220	Predicted protein
A8JDA2	XP_001700391	Predicted protein
A8JDG6	XP_001700473	SR protein factor
A8JDM7	XP_001700552	Flagellar associated protein
Q42690	XP_001700659	Fructose-1,6-bisphosphate aldolase
A8I5N5	XP_001700759	Tetrapyrrole-binding protein
Q8VZX3	XP_001700765	Ubiquitin-conjugating enzyme E2
A8I7D9	XP_001700869	Hypothetical protein
A8I531	XP_001700902	Magnesium chelatase subunit D
A8I5S5	XP_001700943	Hypothetical protein
A8JE81	XP_001701106	Hypothetical protein
Q84TR5	XP_001701111	Microtubule plus-end binding protein

A8JE91	XP_001701113	Chaperonin 60B1
A8JE97	XP_001701117	Predicted protein
A8JEB6	XP_001701130	Hypothetical protein
A8JEE6	XP_001701147	Hypothetical protein
A8JEC1	XP_001701149	Hypothetical protein
A8JEG6	XP_001701174	Hypothetical protein
A8JEJ5	XP_001701186	Vacuolar sorting receptor
A8JES1	XP_001701310	Mitochondrial grpE-type co-chaperone of the HSP70 system
A8JEU4	XP_001701326	Heat shock protein 70A
A8JEV1	XP_001701331	Oxygen evolving enhancer protein 3
A8JF07	XP_001701375	Predicted protein
A8JFA1	XP_001701480	Predicted protein
A8JFB1	XP_001701485	Porphobilinogen deaminase
A8JFG7	XP_001701512	Predicted protein
A8JFL8	XP_001701599	Predicted protein
A8IA98	XP_001701651	Predicted protein
A8I7T8	XP_001701685	Binding protein 1
A8I8Z1	XP_001701760	6,7-dimethyl-8-ribityllumazine synthase
A8I9E8	XP_001701792	Flagella associated protein
A8IA45	XP_001701846	Vacuolar ATP synthase subunit B
A8IAN1	XP_001701881	Transketolase
A8I8A3	XP_001701911	Plastid-specific ribosomal protein 3
A8I8M2	XP_001701933	Predicted protein
A8I8T3	XP_001701945	SNF2 superfamily protein
O49822	XP_001701947	Ascorbate peroxidase
A8I9C7	XP_001701983	Peptidyl-prolyl cis-trans isomerase, FKBP-type
A8I9D5	XP_001701984	Predicted protein

A8I9I9	XP_001701993	Rieske ferredoxin
A8JFN8	XP_001702005	Predicted protein
A8JFR9	XP_001702039	Acetyl CoA synthetase
A8JFZ7	XP_001702110	Hypothetical protein
A8JG54	XP_001702188	Predicted protein
A8JG73	XP_001702217	Flagellar associated protein
A8JG74	XP_001702218	Hypothetical protein
A8JGJ1	XP_001702344	Hypothetical protein
A8HX38	XP_001702347	Eukaryotic translation elongation factor 1 alpha 2
A8JGM1	XP_001702364	Rhodanese-like Ca-sensing receptor
A8IBF4	XP_001702511	Predicted protein
A8IBV4	XP_001702538	Predicted protein
A8ICC8	XP_001702577	Inorganic pyrophosphatase
A8ICE4	XP_001702580	Plastid ribosomal protein L11
A8ICV4	XP_001702611	Photosystem I 8 kDa reaction center subunit IV
A8ID43	XP_001702633	Predicted protein
A8ID55	XP_001702637	Basal body protein
A8IBU6	XP_001702713	Nuclear ribonucleoprotein
A8JGW2	XP_001702816	Cytochrome b6f complex subunit V
A8JGX7	XP_001702846	DnaJ-like protein
A8JH12	XP_001702884	Predicted protein
A8JH68	XP_001702952	Plastocyanin, chloroplast precursor
A8JH98	XP_001702971	Enolase
A8JH97	XP_001702993	Predicted protein
A8JHJ9	XP_001703087	Predicted protein
A8JHN0	XP_001703141	Predicted protein
Q6EMK7	XP_001703164	Chloroplast-targeted RNA-binding protein

A8JHX9	XP_001703215	Elongation factor 2
A8JHX0	XP_001703230	PCNA proliferating cell nuclear antigen
Q9M6B0	XP_001703258	Coiled-coil protein associated with protofilament ribbons
A8IDP6	XP_001703420	Calmodulin
A8IDQ4	XP_001703422	Hypothetical protein
A8IDS5	XP_001703427	Hypothetical protein
Q84X71	XP_001703461	Hypothetical protein
Q84X77	XP_001703484	Predicted protein
Q84X79	XP_001703492	CR008 protein
A8JI94	XP_001703679	Ribosomal protein L22
A8JIB7	XP_001703692	Chaperonin 60A
A8JIB8	XP_001703693	RNA binding protein
A8JIE0	XP_001703710	Chaperonin 20
A8JIE5	XP_001703723	Ribosomal protein S29
A8JII9	XP_001703774	Flagellar associated protein
A8IIA8	XP_001690346	Hypothetical protein
